# Anti-cancer effect of nano-encapsulated boswellic acids, curcumin and naringenin against HepG-2 cell line

**DOI:** 10.1186/s12906-023-04096-4

**Published:** 2023-07-29

**Authors:** Sally Elnawasany, Yusuf A. Haggag, Shahinaz M. Shalaby, Nema A. Soliman, Amira A. EL Saadany, Marwa A. A. Ibrahim, Farid Badria

**Affiliations:** 1 Tropical Medicine Department, Faculty of Medicine, Tanta University, Tanta, Gharbia, 31111 Egypt; 2 Department of Pharmaceutical Technology, Faculty of Pharmacy, Tanta University, Tanta, Gharbia, Egypt; 3 Department of Pharmacology, Faculty of Medicine, Tanta University, Tanta, Gharbia, Egypt; 4 Medical Biochemistry, Faculty of Medicine, Tanta University, Tanta, Gharbia, Egypt; 5 Histology Department, Faculty of Medicine, Tanta University, Tanta, Gharbia, Egypt; 6 Pharmacognosy Department, Faculty of Pharmacy, Mansoura University, Mansoura, Egypt

**Keywords:** Hepatocellular Carcinoma, Boswellic acids, Curcumin, Naringin, Nanoprecipitation, Apoptosis

## Abstract

**Background:**

liver cancer is one of the most common cancers in the world. So far, there is no gold standard treatment for hepatocellular carcinoma. We conducted this in vitro study to assess the effect of three natural products: Boswellic acids, curcumin and naringin versus corresponding nanoparticles (NPs) on Hep G2 cells proliferation.

**Methods:**

Boswellic acid, curcumin, naringin-loaded NPs were prepared using nanoprecipitation method. Human liver (HepG2) cell line was cultured in Dulbecco’s modified Eagle’s medium (DMEM). The cell growth inhibition and cytotoxicity were evaluated by MTT assay.

**Results:**

Boswellic acid, curcumin, naringin were able to inhibit HepG2 cells proliferation. IC50 at 24 h, 48 h showed significant lower values in NPs versus Free herbs. IC50 values of free Boswellic acids and NPs at 24 h were (24.60 ± 1.89 and 7.78 ± 0.54, P < 0.001), at 48 h were (22.45 ± 1.13 and 5.58 ± 0.27, P < 0.001) respectively. IC50 values of free curcumin and NPs at 24 h were (5.89 ± 0.8 and 3.46 ± 0.23, P < 0.05), at 48 h were (5.57 ± 0.94 and 2.51 ± 0.11, P < 0.05), respectively. For free and naringenin NPs, IC50 values at 24 h were (14.57 ± 1.78 and 7.25 ± 0.17, P < 0.01), at 48 h were (11.37 ± 1.45 and 5.21 ± 0.18, P < 0.01) respectively.

**Conclusion:**

Boswellic acid, curcumin, naringin and their nanoprecipitation prepared nanoparticles suppressed Hep G2 cells proliferation.

**Supplementary Information:**

The online version contains supplementary material available at 10.1186/s12906-023-04096-4.

## Background

Liver cancer is ranked as the fifth most common and the second death causing cancer in the world [1]. Egypt is facing a doubling incidence rate in the last years [2]. The majority of primary liver cancers are hepatocellular carcinoma (HCC) [3]. Chronic viral hepatitis (B and C), alcohol intake and aflatoxin exposure are the most frequent causes of HCC [1]. Persistent hepatic inflammation, hepatocytes necrosis and regeneration together with fibrosis add to the pathogenesis of HCC [4]. Where there is an imbalance between activation and inactivation of pro-oncogenes and tumor suppressor genes [5]. Hence, HCC treatment should involve different targeted approaches [6]. The goal of HCC therapeutic modalities is to prolong survival with better life quality. Surgical resection, transplantation, ablation, trans-arterial chemoembolization [7–9] and the tyrosine-kinase inhibitors sorafenib [10, 11] Lenvatinib [12] and regorafenib [13] are modalities with proven survival benefit. Arterial embolization without chemotherapy, external radiotherapy [14] and radio-embolization have shown anti-tumor activity [15–17] but no definitive proof of survival benefit has been found [18]. Systemic chemotherapy carries toxic side effects with no survival benefit. Agents such as tamoxifen, octreotide, and antiandrogens are completely ineffective [19]. 70% of cases are suffering from tumor recurrence in 5 years [20] Up till now, there is no proven recurrence preventing agents [21]. Complementary and alternative medicine (CAM) is commonly practiced worldwide especially among cancer patients in western countries [22, 23]. *Boswellia serrata (B. serrata)* exerts its action against HCC through different ways, suppression of topoisomerase I & II by a caspase-8 dependent pathway [24–26] acting on ribosomal protein production [27, 28] and inhibition of angiogenesis [29]. Poor oral bioavailability of Boswellia is managed by providing it with a standardized meal [30], addition of anionic drugs to improve uptake [31], lecithin delivery form (Phytosome R); nanoparticle delivery systems and synthetic derivatization [32–34]. Curcumin is a natural yellow lipid-soluble compound from the plant Curcuma Longa [35]. It has antioxidant, anti-inflammatory, anti-carcinogenic, and anti-angiogenic properties [36, 37]. Curcumin inhibited hypoxia-inducible factor-1α, the protein which participates in proliferation, migration, and invasiveness of cell cancer [38, 39]. Induction of apoptosis and suppression of cell proliferation is another cytotoxic mechanism of curcumin through suppression of cell membrane axon enrollment, target transcription gene and nuclear Beta-catenin aggregation [40–42]. Metabolic instability, insufficient absorption, and bioavailability all interfere with curcumin pharmacological effect [43]. Curcumin formulations and synthetic analogs have been tried to overcome these obstacles via its combination with polymeric micelles or nanoparticle-based encapsulation which achieved more stability and bioavailability with more potent anti-cancer effect than free curcumin [44, 45]. Naringenin is a flavonoid [46]. Being a lipophilic, facilitates its intestinal absorption through passive diffusion into enterocytes [47]. Naringenin exhibits cytotoxic effect against HCC through induction of the endogenous antioxidant system, inhibition of nuclear factor kappa B (NF-κB), vascular endothelial growth factor (VEGF) and matrix metalloproteinase (MMPs) [48–51]. Furthermore, naringenin up regulated p53 in a dose-dependent manner with subsequent cell cycle arrest in Hep G2 cells [52]. Naringenin attenuates two-pore channels (TPC2) activities and hence, VEGF mediated angiogenesis through interruption of intracellular calcium [53]. Moreover, anti-invasive and anti-migratory action of naringenin were clarified in 12-O-tetradecanoylphorbol-13-acetate (TPA)-activated hepatoma cells by the downregulation of protein kinase C (PKC), epidermal growth factor (EGF), mitogen activated protein kinase (MAPK) and phosphoinositide 3 – kinase L protein kinase B (PI3K/Akt) signaling pathways, and NF-κB, activator protein 1 (AP-1) and matrix metalloproteinase (MMP9) activities [54]. Nanoencapsulation through nanoparticle formation is currently used to increase the oral bioavailability and pharmacokinetic properties of many herbal and non-herbal drugs for the treatment of different diseases, especially cancer treatments [55–58]. These novel nanoparticulate drug delivery systems enhance the biological effects and pharmacokinetics properties of boswellic acids. Therefore, represents a promising method for improving the beneficial therapeutic effects of boswellic acids [59]. Nanoncapsulated curcumin exhibited superior bioactive properties in the aqueous medium, exerting antioxidant and anticancer effects against HCC [60]. The efficiency of Naringenin in treating hepatic cancer was increased by using a nanoparticle drug delivery approach. The enhanced oral bioavailability, and sustained action of entrapped Naringenin, showed better efficacy for the treatment of liver carcinoma [61].

## Materials and methods

This in vitro study was conducted in Tanta University to assess the effect of free Boswellic acids, Curcumin, naringin, and their corresponding nanoprecipitation prepared nanoparticles on Hep G2 cells proliferation.

### Natural products

Boswellic acids, curcumin, [62–64] and naringin [65] were previously isolated in the laboratory of Prof. Dr. Farid Badria (Liver Research Lab, Fab-Lab, Mansoura, university, Mansoura 35,516 Egypt) (Flow charts 1–3, respectively).

### Chemicals and reagents

PLGA (Poly lactic-co-glycolic acid) (Resomer® RG 503 H, 50:50 lactic: glycolic ratio MW34 kDa), Polyvinyl alcohol (PVA, 87–89% degree of hydrolysis, M. wt 31,000–50,000), Tween-80, Phosphate-buffered saline (PBS), Acetone, Ethanol Dichloromethane (DCM) and Acetonitrile (HPLC grade) were purchased from Sigma-Aldrich Chemical Co. (St. Louis, MO, USA). All chemicals and reagents were of analytical/HPLC grade. Milli-Q® water was used throughout the study.

#### Chemicals and reagents

PLGA (Poly lactic-co-glycolic acid) (Resomer® RG 503 H, 50:50 lactic: glycolic ratio MW34 kDa), Polyvinyl alcohol (PVA, 87–89% degree of hydrolysis, M. wt 31,000–50,000), Tween-80, Phosphate-buffered saline (PBS), Acetone, Ethanol Dichloromethane (DCM) and Acetonitrile (HPLC grade) were purchased from Sigma-Aldrich Chemical Co. (St. Louis, MO, USA). All chemicals and reagents were of analytical/HPLC grade. Milli-Q® water was used throughout the study.

### Preparation of drugs-loaded nanoparticles (NPs)

Boswellic acids, curcumin, naringin-loaded NPs were prepared using two different preparation techniques. The first was single oil in water emulsion/solvent evaporation technique (F1) [66]. All drugs (5 mg from each drug) and PLGA (150 mg) were dissolved in 3.5 ml of DCM, once dissolution was complete, the organic solution was added using a syringe to 25 ml of aqueous solution of 1% w/v PVA and sonicated using an ultrasonic homogenizer equipped with a 3.2 mm probe (Cole-Parmer, 4710 series, United States). The final emulsion was stirred overnight to assist in the removal of residual organic solvents. The nanospheres collected by centrifugation were washed three times with ultra-pure water and sucrose solutions. NPs were lyophilized for 72 h using a freeze-dryer and stored at 4 °C until further use. The other preparation method was nanoprecipitation (F2) [67]. Polymer and the three drugs were dissolved in a mixture of acetone/ethanol 3:1 v/v. The drug solution was added to an aqueous solution of 0.5% v/v tween-80 under continuous stirring using a magnetic stirrer for 4 h. The nanospheres were collected using the same procedures, lyophilized, and stored as previously mentioned.

### Physicochemical characterization, particle size, polydispersity index (PDI), and zeta potential (ZP)

Particle size and PDI were obtained by dynamic light scattering technique, while ZP was determined by measurement of the electrophoretic mobility. Each sample was diluted in purified water at 25 ℃. Determinations were performed in triplicate using a Zeta Sizer Nano-ZS (Malvern Instruments Ltd., Malvern, UK).

### Morphological characterization

The morphology of drug-loaded NPs was observed using scanning electron microscopy (FEI Quanta 400 FEG, FEI). A sample of drugs-loaded PLGA NPs was mounted on carbon tape and sputter-coated with gold under a vacuum in an argon atmosphere before microscopic analysis.

### Determination of drug entrapment efficiency (%EE)

Drug encapsulation was measured using the indirect method after collecting the supernatant. The non-encapsulated drug in the supernatant was measured by the High-performance liquid chromatography (HPLC) technique. The HPLC analysis was performed using a C18 column (250 mm×4.6 mm, 5 μm; Waters Corporation, Milford, MA, USA). The mobile phase of curcumin was acetonitrile and water (50:50 v/v) acidified with 2% acetic acid at a flow rate of 1 mL min^− 1^ and UV detection at 425 nm [68]. The mobile phase of naringenin was methanol: water (70:30) acidified by orthophosphoric acid with a flow rate of 1 mL min^− 1^ and ultraviolet detection at 289 nm [69]. The mobile phase used for boswellic acid was acetonitrile and water (85:15) acidified with (0.1% acetic acid) and flow rate of 0.5 mL min^− 1^ and UV detection at 254 nm [70]. The %EE was calculated using the following equation:

$$\%EE=\frac{Total\ Drug\ added-Free\ un-entrapped\ drug}{Total\ Drug\ added}\ \times100$$

### Stability tests

The freshly prepared freeze-dried NPs were stored in a stable chamber at 25 °C for 3 months. Periodically, a sample was collected to measure particle size, PDI, and ZP. The evaluation was performed in triplicate for each analysis.

### Drug release studies

In vitro, drug release test was assessed by direct dialysis method. Drug-loaded PLGA NPs were respectively placed in a 3 000 Da dialysis bag and were incubated in 50 mL of phosphate-buffered saline medium (pH 7.4) [55, 71]. The system was maintained at 37 ± 2 °C, under stirring at 100 ± 10 rpm. Samples were withdrawn at pre-determined intervals and replaced by the same volume of fresh buffer solution. The concentrations of each drug in samples were analyzed by HPLC as described above.

### Herbal nanoparticles codelivery for HPG2 cells cell culture and exposure of NPs

Human liver (HepG2) cells (VACSERA Company, Egypt) were cultured in Dulbecco’s modified Eagle’s medium (DMEM) (Invitrogen, CA, USA) containing 10% fetal bovine serum (Invitrogen), 100 U/mL penicillin, and 100 µg/mL streptomycin (Invitrogen) with the supply of 5% CO2 at 37 °C. At 75-85% confluence, cells were harvested and further subcultured for biochemical studies. Cells were seeded in a 96-well tissue culture plate (4.5 × 10^4^) cells in 100 µl of culture medium. Cells were allowed for 24 h to attach on the surface of the culture plate before exposure to free herbs or codelivery NPs [72]. Dry powder of NPs has been suspended in DMEM at IC 50 of 1 mg/mL. The stock solution was further diluted to different concentrations required for cytotoxicity experiments. The various concentrations of NPs were sonicated at room temperature for 30 min at 40 W to avoid agglomeration before exposure to cells. Cells not exposed to NPs served as a control for each experiment [73].

### Cell viability assay

The Cell growth inhibition and cytotoxicity of free herbs and their codelivery NPs on HepG2 cells were evaluated by MTT assay. The HepG2 cells were treated with different concentrations (1, 3, 5, 7, 9 µg/mL) of free herbs or codelivery NPs for 24 and 48 h. Cell viability was assessed by MTT assay. Live cells could reduce MTT in blue formazan products dissolved in a solvent, and absorbance was recorded at 570 nm employing a microplate reader (Synergy-HT, Biotek, Winooski, VT, USA). Based on the MTT cell viability assay results, the inhibitory concentration (IC 50) values were calculated by GraphPad Prism 8 software (GraphPad S).

### Statistical analysis

All analyses were performed using GraphPad Prism Ver. 6.0 (GraphPad Software Inc. San Diego, CA). All data were expressed as mean ± SEM with statistical significance indicated when *P* < 0.05. Statistical comparisons between control and treatment groups were determined using one-way ANOVA with Tukey’s post hoc tests.

## Results

### Effect of preparation technique on the physio-chemical properties of drug-loaded NPs

**Fig. 1:**
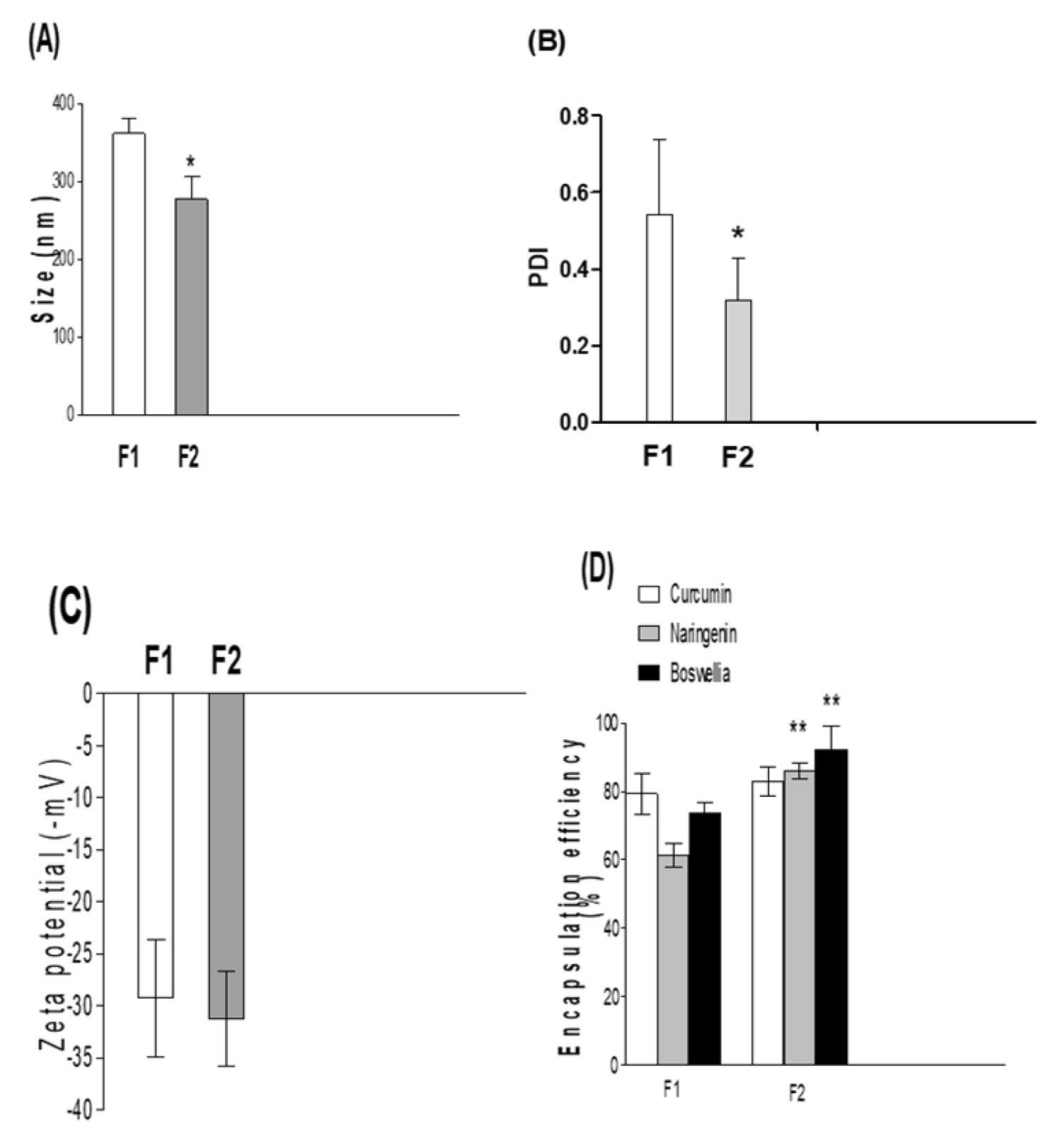
Effect of preparation technique on the physio-chemical properties of drug-loaded nanoparticles. **(A)** polymeric NPs sizes. The figure shows polymeric nanoparticles which were prepared by nanoprecipitation method (F2) were significantly lower in size compared to drug-loaded NP formulations fabricated by O/W single emulsion solvent evaporation technique (F1) **(B)** polydispersity index (PDI) values. The figure shows the size distribution of NPs produced by nanoprecipitation is more uniform and homogenous compared to those prepared by other techniques with significant decrease in polydispersity index (PDI) values. **(C)** zeta potential of both types of NPs. The figure shows zeta potential of both types of NPs exhibited non-significant change. **(D)** drug entrapment efficiency (%EE). The figure shows a sharp increase in %E.E of naringenin and Boswellia was observed in case of NP prepared by nanoprecipitation compared to other NPs. However, %E.E of Curcumin showed non-significant increase

**Fig. 2:**
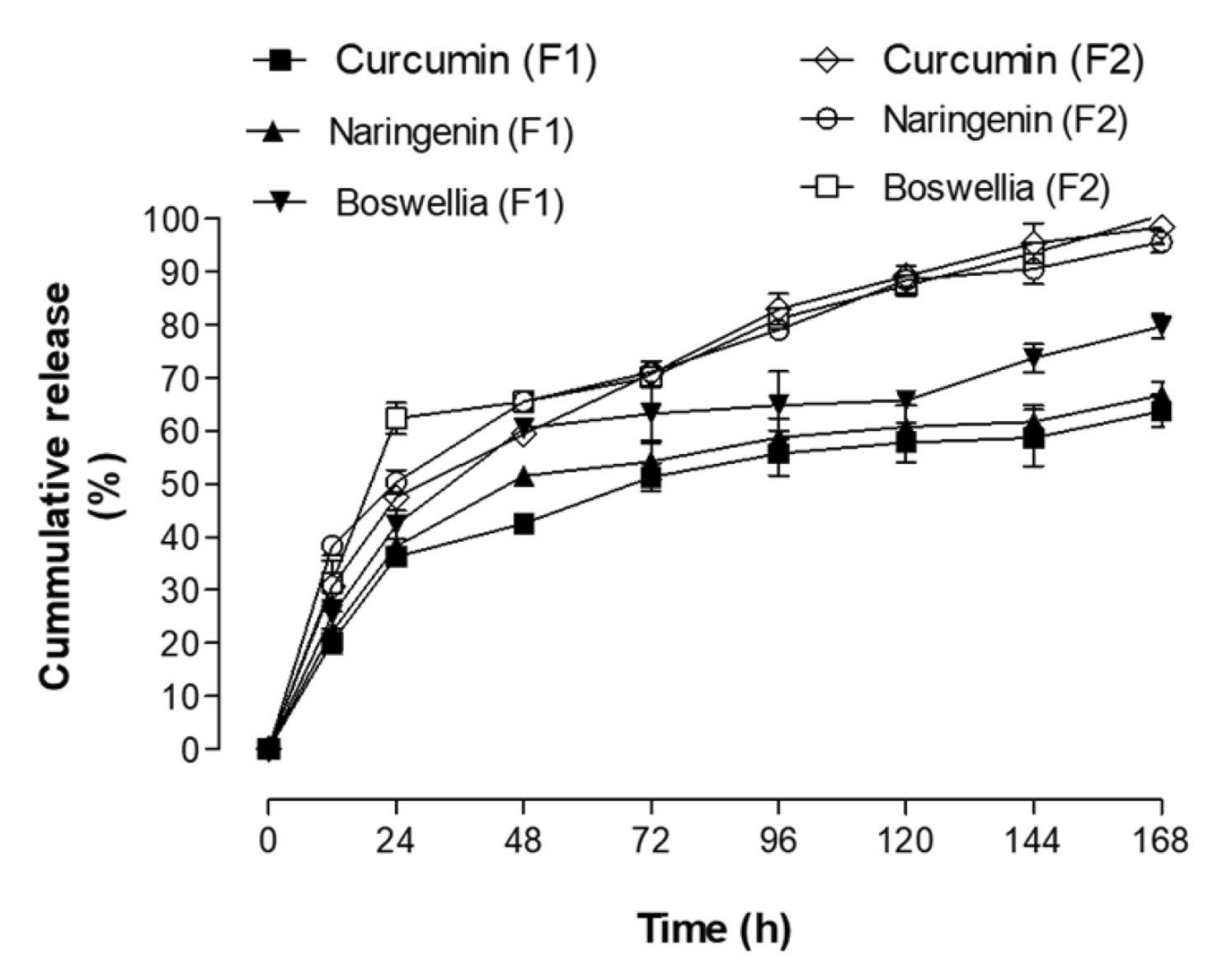
Release profile of both types of nanoparticles. The figure shows a significant increase in initial burst release after 24 h was observed in case of NPs prepared by nanoprecipitation compared to other NPs prepared by emulsion technique

Nanoprecipitation method (F2) prepared polymeric NPs were lower in size and higher in %EE (P < 0.05) compared to O/W single emulsion solvent evaporation technique (F1) prepared NPs (Fig. 1A). The size distribution of NPs produced by nanoprecipitation is more uniform and homogenous than the other technique. There was a significant decrease in PDI values (P < 0.05) (Fig. 1B). zeta potential of both types of NPs exhibited non-significant change (P ˃ 0.05) (Fig. 1C). A sharp increase in %E.E of naringenin and Boswellia (P < 0.01) was observed in case of NP prepared by nanoprecipitation compared to the other NPs. (Fig. 1D). A significant increase in initial burst release (P < 0.05) after 24 h was observed in nanoprecipitation prepared NPs. Initial bursts of 47%, 50% and 62% were observed for curcumin, naringenin and Boswellia versus 36%, 38% and 42% from NPs prepared through emulsion formation, respectively ( Fig. 2). Stability results for three months showed that F2 exhibited a non-significant increase in NP size, PDI also a non-significant decrease in zeta potential was observed which confirmed high storage stability of these lyophilized NPs in vitro (Table 1). From the above results, F2 was superior to F1 consequently, it was chosen for further in vitro studies.

**Table 1:** In Vitro Stability Results of Drug-loaded Nanoparticles (NPs) of Nanoprecipitation Method

Formulation identifier	Time (month)	Size (nm) mean ± SD	PDI mean ± SD	ζ-potential (mV) mean ± SD
F2	0	277.50 ± 29.23	0.319 ± 0.11	-31.23 ± 4.54
	1	286.88 ± 23.31	0.324 ± 0.14	-29.22 ± 3.02
	2	297.15 ± 18.77	0.323 ± 0.17	− 28.97 ± 5.45
	3	307.11 ± 31.72	0.326 ± 0.16	-29.11 ± 6.72

PDI, polydispersity index; ζ-potential, zeta potential

### Scanning electron microscopy

**Fig. 3:**
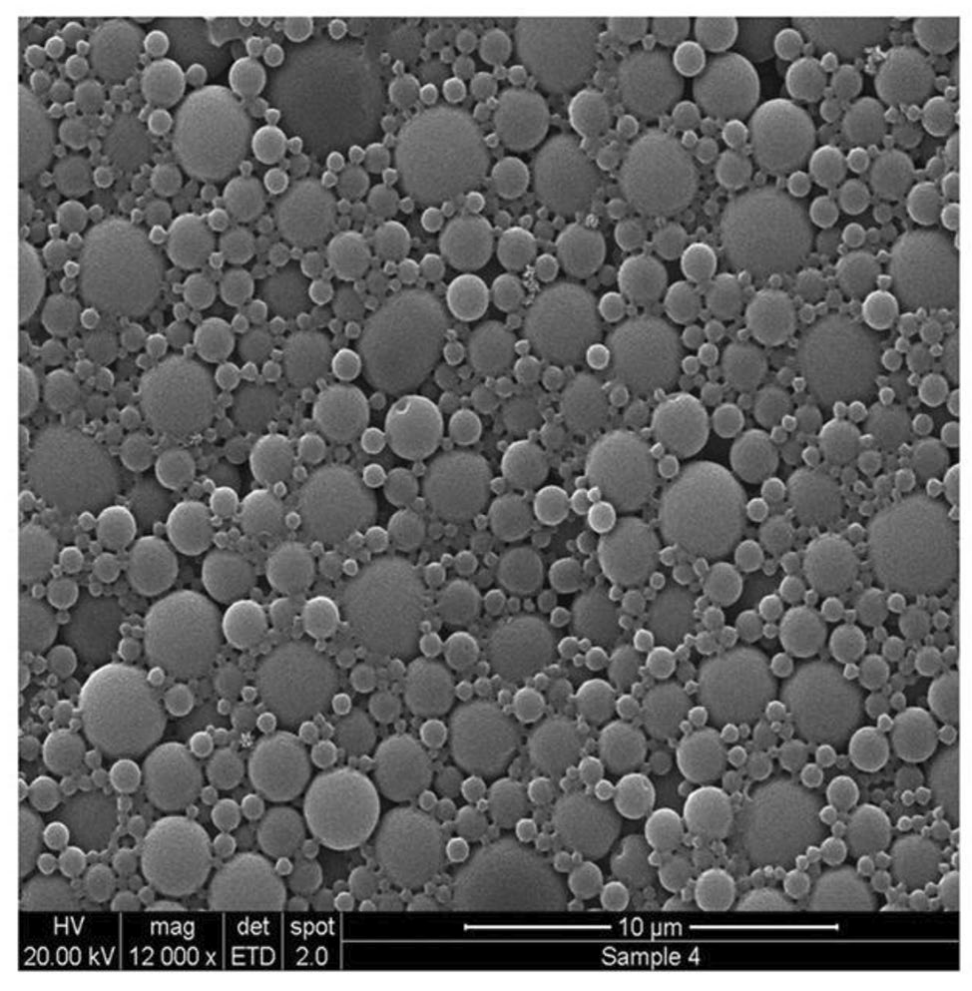
Scanning electron microscopy, a micrograph of the drug-loaded nanoparticles of precipitation method. The figure shows a micrograph of the drug-loaded NP (F2). NP were of spherical shape with a size distribution in approximate agreement with light scattering data. The smooth NP surface was free from void and pores

The drug-loaded NP (F2) were of spherical shape with a size distribution in approximate agreement with light scattering data. The smooth NP surface was free from void and pores (Fig. 3).

### Invitro cytotoxicity assay

Boswellic acids, curcumin and naringenin inhibited proliferation of HepG2 cells with significantly lower IC50 values in NPs (Table 2) .

**Table 2:** Half Maximal Inhibitory Concentration (IC50) of Boswellic acids, Curcumin, Naringenin and Nanoparticles

Mean ± SD	Boswellic acids	Boswellic acids NPs	curcumin	curcumin NPs	naringenin	naringenin NPs
IC50 24 h (µg/ml)	24.60 ± 1.89	7.78 ± 0.54*	5.89 ± 0.80	3.46 ± 0.23*	14.57 ± 1.78	7.25 ± 0.17*
IC50 48 h (µg/ml)	22.45 ± 1.13	5.58 ± 0.27*	5.57 ± 0.94	2.51 ± 0.11*	11.37 ± 1.45	5.21 ± 0.18*

NPs, nanoparticles; * P < 0.05

## Discussion

Hepatocellular carcinoma (HCC) is considered one of the main causes of morbidity and mortality all over the world [74]. Phytochemicals have been utilized in the treatment of variable chronic health problems [75, 76]. There are multiple anti-cancer drugs from natural sources such as vinca alkaloids, vinblastine (VLB), and vincristine (VCR) isolated from the Madagascar periwinkle, Catharanthus roseus G. Don. (Apocynaceae), etoposide (VM 26) and teniposide (VP 16–213) from Podophyllum species (Podophyllaceae), Paclitaxel initially was isolated from the bark of Taxus brevifolia Nutt. (Taxaceae) [77, 78]. As an attempt to introduce some natural agents for HCC treatment, we evaluated the effect of free Boswellic acids, curcumin, and naringenin and nanoparticles on Hep G2 cell proliferation. Single emulsion solvent evaporation technique was evaluated against nanoprecipitation for the entrapment of Boswellic acids, curcumin, and naringenin into PLGA NPs. Generally, the single emulsion process is most suited for water-insoluble drugs such as steroids, while the double emulsion process is considered ideal to encapsulate water-soluble drugs such as peptides [79, 80]. Although the emulsion solvent evaporation technique is the most used method to prepare PLGA NPs, this method is affected by the payload’s physical and chemical properties [81, 82]. In the present study, drug-loaded NPs (F2) prepared by nanoprecipitation resulted in a significant decrease in nano-size and PDI meanwhile a significant increase in %E.E compared to NPs prepared by emulsion/evaporation method (F1). The nanoprecipitation technique was employed instead of the single emulsification/solvent evaporation technique to optimize the physicochemical characteristics of the prepared drug-loaded nanoparticles such as the size, PDI, and %EE [67, 83]. This can be attributed to the ability of these drugs to diffuse through the organic solvent after the emulsification/solvent evaporation step to the water phase leading to inefficient encapsulation of these drugs in the polymeric matrix. On the other hand, the nanoprecipitation technique seems more efficient in increasing the entrapment of all drugs inside the core of the PLGA polymeric matrix. Therefore, the nanoprecipitation method decreased the size and PDI of the prepared NPs and increased all drugs encapsulation, especially for these hydrophobic drugs. The release profile of F2 showed significantly higher initial and higher total amounts of drugs released. The effects of nanoparticles physicochemical properties, such as size, shape and drug encapsulation, on their biological efficacy have been studied extensively. However, the effect of drug entrapment and their controlled drug release on NP efficacy and toxicity has not been thoroughly evaluated both in vitro and in vivo [83]. Our study aims to highlight the importance of nanoencapsulation of three hydrophobic drugs (boswellic acids, curcumin, and naringenin) and their controlled release in vitro compared to these free drugs. The in vitro cytotoxic activity of the drug-loaded NPs was significantly higher than free drugs due to the advantages of cellular interaction between the drug-loaded NPs compared to free drugs. Both free and herbal nanoparticles induced apoptosis in Hep G2 cells with significantly lower IC50 values in nanoparticles (NPs). Boswellia anti-neoplastic action was proved in variable human cancer cell lines and in HepG2 cell line as well [27, 84–86]. Among fractions and volatile oil, Boswellia petroleum ether and methanol extracts were superior with IC50 values of 1.58 and 5.82 µg/mL at 48 h, respectively in comparison to doxorubicin with an IC50 of 4.68 µg/mL at 48 h [87]. Moreover, Boswellia extract inhibited Hep G2 as a monotherapy (IC50 value of 21.21 ± 0.92 µg/mL) and exhibited a synergistic effect in combination with doxorubicin in another study [88]. Curcumin, an active ingredient of *Curcuma longa* possesses multiple chemotherapeutic activities in hepatic, gastric, colon, ovarian, breast and cervical cell cancer [89, 90]. Furthermore, curcumin was superior to other anti-cancer drugs in initiating apoptosis with less insult to normal cells [91, 92]. Change of morphology and inhibition of Hep G2 cell line was induced by curcumin with IC50 of 17.5 ± 3.2 µM. This effect was mediated by interruption in mitochondrial membrane potential and intracellular free Ca2 + level [93]. In a concentration of 50 µmol/L at 24 h, curcumin induced apoptosis in hepatocellular carcinoma in rats by initiation of reactive oxygen species without any harm to normal hepatocytes in a dose and time dependent manner [91]. In another study, Nano curcumin exerted a preventive and therapeutic effect in diethyl nitrosamine (DEN) induced hepatocellular carcinoma (HCC) in rats [94]. Naringenin has a potent effect against multiple cancer cells [52]. Among multiple flavonoids in citrus seeds extract, cytotoxic activity of naringenin on HepG2 cells was revealed. It provoked apoptosis with IC50 value of 172.00 ± 10.39 µM 24 h [95]. Naringenin significantly reduced Hep G2 cells proliferation in dose-dependent manner after 24 h of incubation with IC50 of 100 µM. Furthermore, it increased p53, Bax, caspase-3 and cytochrome C levels in dose dependent manner and decreased the expression of Bcl-2 in comparison to control [52]. This study points out the importance of Boswellic acids, curcumin and naringenin NPs against hepatocellular carcinoma but because of the financial limitation the anti-cancer mechanism was not explained. In fact, the use of NPs has the advantage to augment herbal effect, improve permeability, decrease clearance and provide herbal retention in hepatocellular carcinoma to reach an optimal efficacy [96]. The promising results encourage more research for new herbal formulations and mechanism interpretation.

## Conclusion

Free and nano-encapsulated boswellic acids, curcumin, and naringenin possessed anti-cancer potential by inhibition of HepG2 cell line proliferation. The nanoprecipitation-prepared particles exerted significantly lower IC50 values. This anti-cancer effect carries hope for patients with hepatocellular carcinoma as a safe, and effective therapeutic agent.

## Electronic supplementary material

Below is the link to the electronic supplementary material.

Supplementary Material 1

## Data Availability

The datasets generated and/or analyzed during the current study are not publicly available due to institutional policy but are available from the corresponding author on reasonable request.
