# Supplementary material for: Anti-cancer effect of nano-encapsulated boswellic acids, curcumin and naringenin against HepG-2 cell line
Source: BMC Complement Med Ther. 2023 Jul 29;23:270. doi: 10.1186/s12906-023-04096-4 (PMC10386659; doi:10.1186/s12906-023-04096-4)

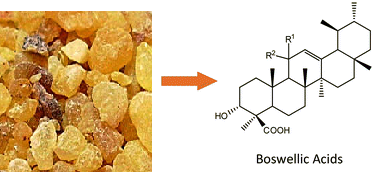


**1. Isolation and characterization of Boswellic acids from oleo-gum resin of *Boswellia carterii***:

**Total resin extract**

**(15 gm)**

**Normal phase Si gel CC**

Pet ether: EtOAc

(Gradient elution)

**(51-70)**

**RP-C18 flash CC**

Methanol:Water

(95:5)

**Silica gel CC**

**Pet ether: EtOAc**

**(9:1)**

**(101-130)**

**(131-160)**

**(161-190)**

**(191-220)**

**(221-250)**

**(251-280)**

**(311-340)**

Fr. 5-11

**Compound A-1**

329 mg

**Preparative TLC**

Pet ether: EtOAc

(9:1)

**Compound A-3**

**30 mg**

**Compound A-4**

**86 mg**

**Crystallization**

**Compound A-5**

**14 mg**

**RP-C18 flash CC**

Methanol:Water

(9:1)

**Fr. 5-6**

**Compound A-6**

**52 mg**

**RP-C18 flash CC**

Methanol:Water

(9:1)

**RP-C18 flash CC**

Methanol:Water

(9:1)

**Fr. 11-12**

**Compound A-7**

**130 mg**

**Fr. 2-3**

**Compound A-8**

**12.1 mg**

**Fr. 10-11**

**Compound A-9**

**28 mg**

**(71-100)**

**RP-C18 flash CC**

Methanol:Water

(8:2)

Fr. 37-41

**Compound A-2**

11.9 mg

|  | **R_1_** | **R_2_** |  |
| --- | --- | --- | --- |
| **A-3** | Ac | 2 H | Acetyl BA |
| **A-6** | Ac | O | AKBA |
| **A-7** | H | 2 H | BA |
| **A-9** | H | O | KBA |

**A-4 (acetyl-α-Boswellic)**


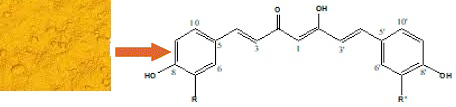


2. **Isolation and characterization of curcumin from turmeric powder of of *Curcuma longa***:

**Turmeric powder**

**(15 gm)**

**Extraction with acetone for 7 hrs at 60 °C.**


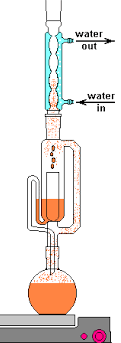


**Cool, remove acetone using Rotary evaporator at 40 °C**

**Orange semisolid residue contains mixture of curcuminoids**

**Dissolve 1 gram from the obtained residue in 5 mL methanol**

**HPLC analysis to determine quantitatively the % of each phenolic compounds in the residue**

**Series of purification steps via Sigel column**


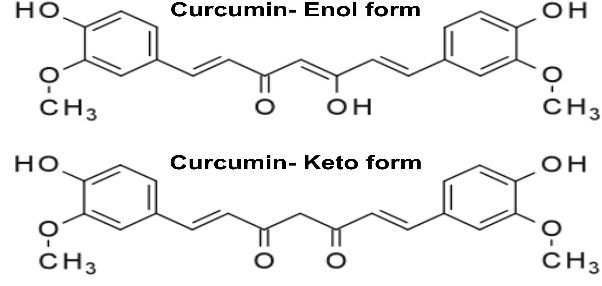


**3. Isolation and characterization of Naringin from Grape fruits "**  ***Citrus*** ***paradisi* "**:


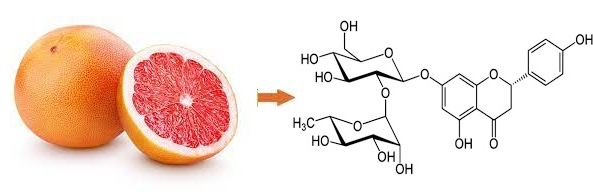


**Fresh Grape"GF" Fruits**

**peels slices and white spongy interior were separated, dried at 40 °C , 30 grams of dried materials were placed in a flask containing 150 mL methanol and set aside at room temperature for 72 hours.**

**Methanolic extract of GF**

**Filter and remove methanol using rotary evaporator under reduced pressure at 40 °C. Add 25 ml of water and stir for about 45 minutes at 65 °C on a hot plate with magnetic stirrer.**

**Crude Mixture containing Naringin**

**5 mL chloroform wad added and the mixture was shaking carefully, separate the organic layer, and filter the obtained crystals and dry in a desiccator.**


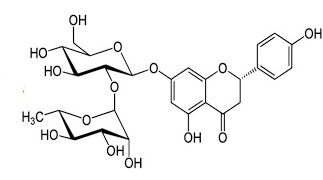

Supplement: Supplementary file 1 — Supplementary Material 1 [file 12906_2023_4096_MOESM1_ESM.docx]
